# Tincture of Iron in Acute Rheumatism

**Published:** 1871-01

**Authors:** 


Tincture of Iron in Acute Rheumatism.—Dr. J. Russell Reynolds reports in the *British Medical Journal* eight cases of acute rheumatism successfully treated by the tinc. ferri chloridi. The pain was relieved very rapidily and convalesence speedily established. In some of the cases the heart was implicated. The quantity given was 50 or 60 drops every six hours.

